# Functional mobility in walking adult population with ataxia of Charlevoix-Saguenay

**DOI:** 10.1186/s13023-021-02054-2

**Published:** 2021-10-14

**Authors:** Isabelle Lessard, Raphaël St-Gelais, Luc J. Hébert, Isabelle Côté, Jean Mathieu, Bernard Brais, Cynthia Gagnon

**Affiliations:** 1 Groupe de recherche interdisciplinaire sur les maladies neuromusculaires (GRIMN), Centre intégré universitaire de santé et de services sociaux du Saguenay–Lac-Saint-Jean, site Jonquière, 2230 de l’Hôpital, C.P. 1200, Jonquière, QC G7X 7X2 Canada; 2 Faculté de médecine et des sciences de la santé, Université de Sherbrooke, Sherbrooke, QC Canada; 3 Centre de recherche Charles-Le Moyne-Saguenay–Lac-Saint-Jean sur les innovations en santé, Université de Sherbrooke, Sherbrooke, QC Canada; 4 Départements de réadaptation et de Radiologie et médecine nucléaire, Faculté de médecine, Université Laval, Quebec, QC Canada; 5 Centre interdisciplinaire de recherche en réadaptation et intégration sociale (CIRRIS), Institut de réadaptation en déficience physique de Québec, Quebec, QC Canada; 6 Montreal Neurological Institute, McGill University, Montreal, QC Canada

**Keywords:** ARSACS, Recessive ataxia, Functional mobility, Walking

## Abstract

**Background:**

This study aimed to describe lower limbs impairments, balance and activity limitations related to indoor mobility in adult walkers with autosomal recessive spastic ataxia of Charlevoix-Saguenay (ARSACS).

**Results:**

Twenty-five participants were recruited with a mean age of 32.2 (± 10.4) years with 45.7% using a walking aid. There is a significant difference between participants with and without a walking aid in terms of lower limbs coordination, balance and mobility. Although participants who walk without a walking aid perform better than the others and they are below predictive or reference values. Despite significant mobility limitations, only mild spasticity and passive range of motion limitations were observed. However, there is a significant difference between unaffected individuals and participants with ARSACS for lower limb muscle cocontraction.

**Conclusions:**

Results show a high level of lower limb impairments, balance and mobility limitation in adults’ participants with ARSACS that are still walking, including people not using a walking aid. One of the most original finding is the presence of excessive cocontraction and a relatively mild level of spasticity in the lower limbs muscles. Results of this study better circumscribes the impairments and activities that should be the focus of intervention including rehabilitation in ARSACS.

## Background

Autosomal recessive spastic ataxia of Charlevoix-Saguenay (ARSACS) [1] is the second most frequent recessive ataxia and caused by mutations in the *SACS* gene on chromosome 13q12 [2, 3]. ARSACS, originally described in 1978, is a progressive disorder mostly present in Quebec (Canada) with a prevalence of 1/1 932 in the Charlevoix and Saguenay-Lac-St-Jean regions due to a founder effect [1, 4]. Symptoms include ataxia and dysarthria, lower limb spasticity and distal weakness [5]. One study had documented disabling cocontractions (or coactivations [6]) of the agonist/antagonist muscles during lower limb movement [7]. A high level of variability in terms of clinical presentations, severity and progression of signs and symptoms has been documented [8].

Mobility issues that start at a young age [5, 8] are clinically amongst those that greatly restrict participation in work, leisure activities and independence at home [9]. For this study, functional mobility [10] is limited to indoor mobility to reduce the impact of environmental factors (external), such as the climat and the topography. In ataxic gait disorders, impaired mobility is an early predictor of activity limitations and associated with falls, decline in independence and quality of life, institutionalization, and death [11–13].

To the best of our knowledge, no studies have ever focused on a comprehensive description of impairments and activity limitations related to indoor mobility in the ARSACS adult walking population. In order to target interventions that maintain physical autonomy as long as possible and increase trial readiness it is important to systematically document key characteristics of this population in their indoor environment. This cross-sectional study aimed to: (1) document lower limb impairments and activity limitations related to indoor mobility in adult walkers; (2) compare physical performances between different mobility stages and with reference values; and (3) explore the associations between age, disease severity and mobility-dependent activity limitations.

## Method

### Participants

Participants were recruited among a subset of 169 patients with ARSACS followed at the Neuromuscular Clinic of the *Centre Intégré Universitaire de Santé et de Services Sociaux* (CIUSSS) *du Saguenay–Lac-St-Jean* (Quebec, Canada). Documentation of lower limb impairments and mobility was part of a larger study on ARSACS natural history (baseline: 2013, phase 2: 2015, phase 3: 2017) (8). At first, 31 walkers who had participated in at least one previous data collection were invited to participate in this third phase. New participants were thereafter recruited using a stratified sampling strategy for sex and age to complete the sample size. Patients meeting the following criteria were included in the study: (1) ≥ 16 years old; (2) genetically confirmed ARSACS diagnosis; (3) walker (indoor walking abilities with or without walking aids); (4) ability to provide informed consent. Patients with other diseases causing physical limitations, with a Baclofen intrathecal pump or pregnant were excluded. For the surface electromyography, nine healthy participants were enrolled as a control group with a sampling strategy by sex and age. The Ethics Review Board of the CIUSSS Saguenay–Lac-St-Jean (Quebec, Canada) approved the study and written informed consent was obtained from all participants.

### Data collection

Participants were assessed over two half-day sessions within a 2-week interval. Outcome measures were conducted in a standardized order and by the same trained physiotherapist using standard operating procedure (SOP). A sociodemographic questionnaire was administered to document information about age, sex, mobility stages (use of a walking aid indoors), and falls. Mobility stages were defined as follows: (1) No walking aid; (2) Walking aid (cane or 2 or 4 wheeled walker). Disease severity was assessed using the Disease Severity Index for Autosomal Recessive Spastic Ataxia of Charlevoix-Saguenay (DSIARSACS). This tool is an eight items ordinal scale with a total potential score of 38 [14]. The lower the score, the lower the disease severity. Cerebellar ataxia was also quantified using the Scale for the assessment and rating of ataxia (SARA), which includes eight items for a total score between 0 (no ataxia) and 40 (most severe ataxia) [15].

### Outcome measures

#### Lower limb impairments

Spasticity was assessed with the modified Ashworth scale in a supine position. Mild spasticity was defined as grades 1 and 1+, moderate spasticity as grades 2 and 3, and severe spasticity as grade 4 [16, 17]. Hip adductor, knee flexors and extensors and ankle plantar flexors muscles were evaluated.

The passive range of motion of lower limb joints was measured bilaterally. Participants were in the following positions: sitting down for hip external rotation; supine lying for hip abduction, knee flexion and extension, and ankle dorsiflexion; and side lying for hip extension. Each joint was moved passively to its full extent and endpoint measurements were made using a universal and a digital goniometer (Digital Protractor Series 950-Pro 360, Aurora, USA). Bone markers have been standardized [18].

Coordination of lower limbs was measured using the Lower Extremity Motor Coordination Test (LEMOCOT) [19]. In this test, while sitting down, participants alternatively touch two targets placed 30 cm from each other as fast as possible during 20 s (2 trials). The reliability (intra and interrater) of this test is excellent in ARSACS population, and its construct validity was demonstrated [20].

Lower limb muscle activation was measured using surface electromyography (EMGs). Surface myoelectrics signals were recorded at 2000 HZ using a 16-channel wireless system (Trigno™ EMG, Delsys, MA, USA). After skin preparation, bipolar surface electrodes were placed over the muscle in the direction of the muscle fibers according to the European recommendations for surface electromyography (SENIAM) [21]. Two electrodes were placed on the right leg on the rectus femoris and biceps femoris. Two tasks were evaluated: knee flexion with participant on ventral decubitus and knee extension in sitting position (open chain movement). The mean of three trials for each task was used for analyses.

#### Balance and mobility

Balance and risk of falling were evaluated with the Berg Balance Scale (BBS) [22]. The BBS is a 14-item ordinal scale graded from 0 to 4 (for a potential total score of 56 [best performance]). Its construct validity was also demonstrated in ARSACS [23].

*Activities-specific Balance Confidence-simplified (ABC-Simplified).* Participants answered a questionnaire to rate their perceived degree of balance confidence (0 to 100%) relating to 16 daily activities, such as: walking around the house or going up and down the stairs [24].

Short distance walking speed was assessed with the 10-Meter Walk Test (10mWT) at self-selected and maximal speed (1 trial for each). These tests measure the time required to cover a 10-metre distance. The 10mWt has excellent interrater reliability, and construct validity was supported in the ARSACS population [23].

The ability to get up and sit on a chair without using the upper limbs was assessed using the 30-Second Chair Stand test (30 s-CST) [25]. The number of full sit-to-stands correctly performed in 30 s was recorded. The mean of two trials was used for analyses.

The Timed Up & Go test (TUG) was used to measure functional mobility (sit to stand from a chair, short distance walking and change of direction) [26]. The TUG consists of self propelling out of a chair, walking 3 m at self-selected speed, turning around on the line, walking back to the chair and sitting down. The time to perform the task is recorded and the mean of three trials was used for analyses. The TUG has demonstrated strong construct validity in the ARSACS population [23].

### Statistical analysis

Descriptive statistics were used for continuous variables (mean, median, SD, ranges), and frequency and percentage for categorical variables. For continuous variables, the Kolmogorov-Smirnov test was used to analyze the normality of the data. To compare the performance and characteristics of participants between mobility stages, a Student’ s t-test (Mann-Whitney U-test) or a Chi-Square test was used. For conciseness and due to the lack of significant statistical differences, results are shown for the right side only for ranges of motion and LEMOCOT. The interaction between self-selected and maximal walking speed and subgroup effects (mobility stages) was analyzed using mixed between–within participants repeated measures analysis of variance. A significant interaction within-participants indicates a difference in the ability to walk faster, i.e. the delta between maximal and self-selected walking speed is different between subgroups (mobility stages). A significant interaction between participants indicates that the mean score obtained for each subgroup was different. Participants’ results were expressed as a percentage of the predictive or reference values from LEMOCOT [27], 10mWT [28] and passive ranges of motion for lower limb joints [29]. The percentage of the reference or predictive values for the 10mWT and the LEMOCOT were compared between mobility stages and sex using a Student’s t-test (Mann-Whitney U-test). For the BBS, a cut-off score of < 45 was used to determine which individuals had an increased risk of falling [30]. The percentage of participants with a result below 45 was compared to the percentage of participants with a result of 45 or more using the Chi-Square Test. Correlation coefficients (Spearman ρ, Pearson *r*) were used to assess associations between the scores of the TUG, 10mWT (self-selected speed), 30 s-CST, and the participant’s age and disease severity (SARA and DSI-ARSACS).

The EMG data were processed using a MATLAB software (version 9.3.0.713579, MathWorks, Natick, MA, USA). The raw EMG signals were analog processed with a differential amplifier (bandwidth = 20 ± 5–450 ± 50 Hz, signal range 11 mV, common mode rejection ratio > 80 dB at 60 Hz, baseline noise < 0.75 µV, input impedance > 10 Ω and sampling rate = 1925.93 samples/s). EMG signals were digitally filtered off-line with a zero lags fourth-order Butterworth filter (band-pass 20–450 Hz). Thereafter, a root mean square (rectangular window of 20 ms) for rectifying and smoothing the signal was applied. For each task, the participant EMG signals for each muscle were normalized in time. A cocontraction index (CI) [31] was obtained by calculating the area under the curves as follows:

$$\begin{aligned} {\text{Knee}}\;{\text{flexion: CI}} & = {\text{rectus}}\;{\text{femoris}}\;{\text{EMGs/biceps}}\;{\text{femoris}}\;{\text{EMGs}} \\ {\text{Knee}}\;{\text{extension: CI}} & = {\text{biceps}}\;{\text{femoris}}\;{\text{EMGs/rectus}}\;{\text{femoris}}\;{\text{EMGs}} \\ \end{aligned}$$

To compare CI of ARSACS participants across mobility stages and with healthy participants, an ANOVA test was used with Tuckey test for post-hoc analyses (group 1: healthy; group 2: ARSACS without walking aid; group 3: ARSACS with walking aid) for knee flexion and extension.

The power of the study was calculated using the Two-Sample t Test for Mean Difference using results of the 10mWT self-selected speed. For all tests a *p* value < 0.05 was considered significant. Data were analysed using IBM SPSS Statistics for MAC, Version 20.0 (Armonk, NY: IBM Corp).

## Results

### Demographic and clinical characteristics

From the 169 ARSACS patients followed at the neuromuscular clinic, 50 people met the inclusion criteria, 11 refused to participate (mean age: 32.2 years, 45.5% were men) and four were not contacted since a sufficient number of participants had been recruited for the larger project. A total of 35 participants were recruited among whom 16 participants (45.7%) used a walking aid (cane [3], 4-Wheel Walker [10] and 2-Wheel Walker [3]). Of these 35 participants, 30 were homozygous for the 8844deIT mutation, two were compound heterozygotes for the 4744G > A and the common 8844 delT mutations, two were compound heterozygotes for the 7504 C > T or the 5836T > C and the common 8844 delT mutations, and one was homozygous for the 7504 C > T mutation. A total of eight (22.9%) participants took Baclofen daily (no walking aid [3], walking aid [5]). Table 1 shows the participants’ characteristics. As a control group for the EMG analyses, nine healthy participants were recruited (mean age: 36.4 years, 44.4% were men).

**Table 1:** Characteristics of the study population (n = 35)

Characteristic	Total group n = 35	No walking aid n = 19	Walking aid n = 16	*p*-value^*^
*Age, (y)*				
Mean (SD)	32.2 (10.4)	26.5 (7.6)	38.9 (9.4)	**< 0.001**
Range	16–65	16–42	25–65	
*Sex, n (%)*				
Men	15 (42.9)	11 (73.3)	4 (26.7)	0.087
Women	20 (57.1)	8 (40.0)	12 (60.0)	
*Age groups, n (%)*				
16–29	14 (40.0)	13 (92.9)	1 (7.1)	
30–39	16 (45.7)	5 (11.0)	11 (68.8)	–
≥ 40	5 (14.3)	1 (20.0)	4 (80.0)	
*Falls (period)*				
6 months				
Mean (SD)	8.8 (11.1)	11.1 (13.5)	6.3 (7.3)	0.219
Range	0–50	0–50	0–26	
*1 year*				
Mean (SD)	17.6 (22.1)	21.6 (26.9)	13.0 (14.4)	0.262
Range	0–100	0–100	0–52	
*Disease severity*				
DSI-ARSACS (/38)				
Mean (SD)	13.5 (4.3)	10.8	16.7	**< 0.001**
Range	4.5–22.0	4.5–16.0	11.0–22.0	
SARA (/40)				
Mean (SD)	13.4 (5.9)	9.2 (4.0)	18.5 (3.0)	**< 0.001**
Range	4.0–22.5	4.0–20.0	11.0–22.5	

*Statistical difference between mobility stages

Bold indicates *p* value < 0.05

### Lower limb impairments

No participant exhibited severe spasticity in any of the four muscle groups assessed (see Table 2). More than 50% of participants had mild spasticity for hip adductor and knee flexor muscles, and only 11.4% had moderate spasticity for the knee extensor muscles. The lower limbs’ passive ranges of motion are detailed in Table 3. All passive joint ranges of motion are close to reference values with the exception of hip extension, which is greatly diminished. There were no significant differences between mobility stages for all lower limbs’ passive ranges of motion.

**Table 2:** Spasticity level in the lower limbs

Spasticity	Sample	None n (%)	Mild n (%)	Moderate n (%)	*p*-value***
Hip adductors	Total (n = 34)	13 (38.2)	20 (57.1)	1 (2.9)	–
	NWA*	8 (47.1)	9 (52.9)	0 (0)	0.388
	WA**	5 (29.4)	11 (64.7)	1 (5.9)	
Knee extensors	Total (n = 34)	20 (58.8)	10 (29.4)	4 (11.4)	–
	NWA	10 (58.8)	6 (35.3)	1 (5.9)	0.497
	WA	10 (58.8)	4 (23.5)	3 (17.6)	
Knee flexors	Total (n = 34)	15 (44.1)	19 (55.9)	0 (0)	–
	NWA	9 (52.9)	8 (47.1)	0(0)	0.300
	WA	6 (35.3)	11 (64.7)	0 (0)	
Ankle plantar flexors	Total (n = 31)	20 (64.5)	11 (35.5)	0 (0)	–
	NWA	12 (80.0)	3 (20.0)	0 (0)	0.135
	WA	8 (50.0)	8 (50.0)	0 (0)	

*NWA: No walking aids

**WA:Walking aids

***Statistical difference between mobility stages

**Table 3:** Lower limbs’ passive range of motion

	Total sample (n = 35)	% of the reference values	No walking aid (n = 19)	Walking aid (n = 16)	*p*-value^*^
*Hip*					
Abduction	25.5 (9.8)	NA	24.9 (7.8)	25.5 (11.9)	0.797
Extension	1.9 (5.3)	10.4 (29.7)	1.6 (5.8)	1.9 (4.7)	0.658
External rotation	36.0 (4.2)	NA	37.4 (9.4)	34.2 (7.4)	0.377
*Knee*					
Flexion	147.5 (5.9)	105.3 (4.5)	147.8 (5.5)	147.6 (6.6)	0.629
Extension	2.9 (4.2)	100.8 (2.3)	1.6 (3.6)	3.6 (3.5)	0.092
*Ankle*					
Dorsiflexion	8.2 (5.3)	94.9 (5.3)	9.0 (6.1)	7.5 (4.4)	0.226

*Statistical difference between mobility stages

**Results are presented in degrees as Mean (standard deviation)

*NA* Not available

According to lower limb coordination, the mean number of touched targets in the LEMOCOT test was 24.6 (± 10.6), but results vary significantly from one individual to another (range: 3–53). The mean number of touched targets for participants without a walking aid was higher compared to those using a walking aid (29.9 [± 9.9] and 17.9 [± 7.1], respectively: *p* < 0.001). There was a difference in lower limbs’ muscles CI for the three groups in knee flexion (F [2.37] = 7.4, *p* = 0.002) and knee extension (F [2.36] = 6.4, *p* = 0.004). Results indicated that only the mean score for the healthy group was different from ARSACS participants using a walking aid regarding CI in knee flexion (*p* = 0.001) and knee extension (*p* =  0.003). The lower limbs’ muscles CI are detailed in Table 4.

**Table 4:** Lower limbs’ muscles cocontraction index

	Healthy	ARSACS			*p*-value
		Total sample	No walking aid	Walking aid	
* Knee flexion*	*n = 9*	*n = 32*	n = 17	n = 15	**0.002**
	0.12 (0.07)	0.37 (0.23)	0.31 (0.18)	0.43 (0.26)	
min–max	0.05–0.27	0.13–0.98	0.12–0.82	0.15–0.98	
* Knee extension*	*n = 9*	n = 31	n-17	n = 14	**0.004**
	0.21 (0.10)	0.50 (0.31)	0.42 (0.15)	0.58 (0.41)	
min–max	0.07–0.34	0.09–1.63	0.09–0.67	0.10–1.63	

*Results are presented as Mean (standard deviation)

Bold indicates *p* value < 0.05

### Activity limitations

Results for the total sample and for each mobility stage are shown in Table 5. For every outcome measure of mobility and balance, there is a difference between participants with and without a walking aid. An effect of mobility stages was observed regarding the ability to walk faster (Δ 10mWT = maximal speed – self-selected speed) (*p* =  0.040). Indeed, results showed that participants not using a walking aid have a higher ability to walk faster (0.36 [± 0.26]) compared to those using a walking aid (0.20 [± 0.12]). With a significance level at α = 0.05, the power of the test to detect a difference of 0.53 m/s at the 10mWT self-selected speed is > 0.999 for samples size of 19 participants who walk without a walking aid and 16 participants who walk with a walking aids with means of 1.08 m/s and 0.55 m/s and standard deviation of 0.32 m/s and 0.12 m/s, respectively. Overall, 23.5% of participants using a walking aid were unable to perform a sit-to-stand transfer without using their upper limbs.

**Table 5:** Performance comparison in clinical variables of mobility and balance between mobility stages

	Total	No walking aid	Walking aid	*p*-value^*^
	n = 35	n = 19	n = 16	
*10 mWT self-selected speed (m/s)*	0.84 (0.36)	1.08 (0.32)	0.55 (0.12)	**< 0.001**
min–max	0.29–1.46	0.44–1.46	0.29–0.74	
*10mWT maximal speed (m/s)*	1.13 (0.49)	1.43 (0.43)	0.73 (0.17)	**< 0.001**
min–max	0.45–2.17	0.66–2.17	0.45–1.10	
*TUG (s)*	17.02 (7.59)	12.17 (5.13)	22.85 (5.74)	**< 0.001**
min–max	6.90–35.23	6.90–25.27	12.89–35.23	
* 30 s-CST (no. of rep)*	6.77 (5.38)	10.72 (3.65)	2.31 (2.93)	**< 0.001**
min–max	0–16.0	1.5–16.0	0–9.5	
*BBS (/56)*	34.71 (15.52)	45.56 (10.70)	22.50 (10.0)	**< 0.001**
min–max	6–56	11–56	6–45	
*ABC scale (/100)*	71.99 (14.02)	80.25 (11.33)	62.07 (10.03)	**< 0.001**
min–max	40.0–97.8	55.6–97.8	40.0–77.8	

*Statistical difference between mobility stages

**Results are presented as mean (standard deviation), range

Bold indicates *p* value < 0.05

Comparison of results with reference values (LEMOCOT) and predictive values (10mWT self-selected) are shown in Table 6, as well as the number of participants at high risk of falling (BBS result below 45). There were no significant differences between sex for the mean percentage of the predictive or reference values for the 10mWT self-selected and the LEMOCOT. Table 7 shows the level of association of participant’s age and disease severity and mobility outcomes measures.

**Table 6:** Comparison with reference values

	Total	No walking aid	Walking aid	*p*-value***
	n = 35	n = 19	n = 16	
LEMOCOT, mean (SD)*	52.3 (21.6)	63.4 (20.1)	40.1 (16.6)	**0.002**
10mWT, mean (SD)*	66.6 (29.0)	85.8 (25.6)	43.9 (9.8)	**< 0.001**
BBS, n (%)**	22 (62.9)	7 (36.8)	15 (93.8)	**< 0.001**

*% of the predictive or reference values

**Number of participants at higher risk of falling (BBS result < 45)

***Statistical difference between mobility stages

Bold indicates *p* value < 0.05

**Table 7:** Association between participant’s age and disease severity and mobility outcomes measures

		10mWT self-selected	10mWT maximal speed	TUG	30 s-CST	ABC scale	BBS
Age		− 0.641*	− 0.656*	0.673*	− 0.587*	− 0.370**	− 0.628*
*Disease severity*							
DSI-ARSACS		− 0.760*	− 0.830*	0.821*	− 0.820*	− 0.611**	− 0.753*
SARA		− 0.788*	− 0.797*	0.884*	− 0.929*	− 0.684*	− 0.929*

*Correlations are significant (*p* < 0.001)

**Correlations are significant (*p* < 0.005)

## Discussion

This is the first study to document impairments and activity limitations related to indoor mobility using quantitative assessment among a large cohort of adult walkers with ARSACS. Results illustrate the high level of variability within a specific mobility stage regarding disease severity. Only a few studies quantified the disease severity and mainly in small cohorts. When comparing our results with those obtained in other ARSACS populations, the mean disease severity as assessed by the SARA is similar for people who walk with a walking aid to the result obtained by Vermeer et al. (5/16 participants) within the same age range (SARA mean score = 16.7; ranging from 14 to 19.5) [32]. To the best of our knowledge, there is no comparative data for disease severity in cohorts of adult with ARSACS who walk without using a walking aid.

According to lower limb impairments, our study has only documented a low level of spasticity, despite the obvious limitations of our cohort in terms of walking and transfers. Also, no significant difference on the level of lower limbs’ spasticity between mobility stages was observed. These findings are consistent with those obtained by Krygier et al. [33], who noted that despite the loss of walking abilities, people with ARSACS only had a low level of spasticity in lower limbs. In a retrospective study, the lower limb muscles’ spasticity at the age of 18 has been reported to be mild for 84% and moderate for 10% of people with ARSACS [17]. The daily intake of Baclofen by eight participants may have had an impact on the level of spasticity assessed. However, the prescription and the dosage are mainly to decrease the impact of neurogenic bladder and the presence of spasms in the lower limb. Only few studies have quantified spasticity in lower limb muscles using a standardized protocol for speed, angle of assessment, positioning and rating [1, 17, 33, 34]. Furthermore, the modified Ashworth scale is used worldwide in research and clinical follow-up but the interrater reliability is poor in ARSACS population [35]. Because of the lack of a standardized protocol and the poor psychometric properties of the modified Ashworth scale, it is in fact difficult to properly assess spasticity and compare results between studies.

This is the first study that used quantified measures to evaluate lower limb passive joint range of motion in ARSACS population. There was no significant difference between adults with ARSACS who walk with or without a walking aid. We documented significant limitations in passive range of motion for the hip (mainly extension) in young adult walkers without a walking aid. Indeed, they obtained values representing 10% of the reference values (mean: 1.9° [± 5.3°]). In order to allow a good propulsion in walking, at the end of the support phase, a range of motion of 10 degrees of hip extension is required [36]. The hip joint retractions certainly have an impact on the kinetics and kinematics parameters of walking, which could be the subject of future studies. Only small limitations in passive range of motion were noted at the knee and ankle joints in comparison to reference values. It is clinically recognized that knee and ankle joint retractions appear gradually as the disease progresses [34]. It is therefore possible to hypothesize that these retractions are more common for patients who use a wheelchair on a daily basis.

This study demonstrated that people who do not use a walking aid already present a significant decrease of lower limb coordination in comparison to reference values. Lower limb ataxia is one of the main impairments for people with hereditary ataxia due to the degeneration of the cerebellum. This impairment likely contributes greatly to walking difficulties, as observed in other forms of ataxia [37, 38]. One of the novel features of this study was the exploration of the lower limbs’ muscular activation. We documented that the lower limb muscle cocontraction index is 2.38 times higher in people with ARSACS compared to healthy people for active knee extension, and 3.08 times higher for active knee flexion. As shown by the CI reported in Table 4, during an active knee extension, while healthy participants are activating their antagonists muscle (knee flexors) at a level of 21% of the agonists’ level, people with ARSACS are activating their knee flexors at a level corresponding to 50% of the knee extensors, suggesting an abnormal level of antagonists muscle activity. Similarly, during an active knee flexion, while healthy participants are activating their antagonists (knee extensors) at a level of 12% of the agonists’ level, the people with ARSACS are activating their knee extensor at a level corresponding to 37% of the knee flexors. We noted no significant differences between adults with ARSACS who walk with or without a walking aid, but there was a significant difference between healthy people and participants with ARSACS who use a walking aid. Our results also illustrate the high level of variability within mobility stage and from one stage to another in regard to lower limbs’ muscles cocontraction index. Since gait varies a lot amongst people with ARSACS who carry the same genetic mutation, it would be interesting to eventually determine if the increase in the lower limbs’ muscles cocontraction affects only one knee movement (flexion or extension) or both. It could also be interesting to study the lower limbs’ muscles cocontraction while walking. Only one study had explored the lower limbs’ muscles cocontraction in a few people with ARSACS in 1980 [7]. Despite methodological differences between these two studies, the results obtained are in fact similar. Increased muscle cocontraction in neurological disorders, such as spastic paraparesia or parkinson, can be interpreted as a primary deficit due to impaired reciprocal inhibition and/or a lack of selectivity in the command muscle drive and/or a as an attempt to reduce instability in the lower limbs during walking [39, 40]. In hereditary ataxia increased muscle cocontraction is related to compensatory strategy rather than a primary deficit of the neuromuscular system [41]. In ARSACS that could also be related to central nervous impairment (cerebellum and pyramidal tract). The presence of an excessive muscle cocontraction could be perceived as spasticity during the administration of other clinical tests.

People with ARSACS have a very high risk of falling. Indeed, our results have showed that walkers with or without a walking aid fall on average 18 times a year. Van de Warrenburg and al. [42] reported that falls are very common in patients with cerebellar ataxias and the consequences of these falls can be serious and lead to injuries or a fear of falling. In their study, almost 93% of patients reported one or more falls in the past 12 months. As indicated by the BBS score, 36.8% of patients without a walking aid and 93.8% of those with a walking aid are at high risk of falling (BBS score < 45) while walking, transferring or simply standing up without support. In our cohort, the number of self-reported falls for periods of six month and a year are considerably lower for walker using a walking aid inside the house.

Clinically, it is well known that a large proportion of people with ARSACS do not want to use a walking aid at home even though the severity of their physical impairments and activity limitations would indicate that they need one. If we look closer at the results of the BBS, it is possible to note that despite the high risk of falling, seven participants in our cohort were not using a walking aid at home. In particular, one participant who had a very low BBS score (11/56) and did not use a walking aid at home fell 50 times in six months, which reflects the very high risk of falling and the need to use a walking aid to prevent future injuries. As clinicians lack data about both the presentation and the progression of ARSACS population’s impairments, they are unable to recommend appropriate preventive measures or increase the adoption of walking aids for daily use, such as 4-wheel walkers or wheelchairs. Since people with ARSACS also tend to have some cognitive rigidity, embracing changes can be a challenge [43]. This resistance to change could explain some of the extreme results of our study and would certainly be a great aspect to explore deeper in further studies.

Activities related to indoor mobility included abilities to walk and make a sit-to-stand transfer. In our cohort of walkers, we noted that the proportion of people over 30 years who use a walking aid is higher than those in their twenties. People over 40 who maintain their walking ability do so mainly with a walking aid, an observation also reported in other studies [32, 33]. Results also show that ARSACS leads to a significant decrease in self-selected walking speed compared to the predictive values, mainly for walkers with a walking aid. Those results were also obtained in several populations with hereditary ataxias [44]. This lower walking speed combined with a more limited ability to walk faster, especially for walkers with a walking aid, may exacerbate the impact of some associated impairments that require the ability to move quickly, such as urinary urgency or bladder incontinence [45]. This is important as such impairments are strongly associated with risk of fall in older adults [46].

Our results also show a significant difficulty in performing the sit-to-stand transfer without using the upper limbs, even for people who do not use a walking aid. In fact, the results obtained for walkers without walking aids are similar to those obtained for healthy people over 85 [47]. Overall, 23.5% of participants using a walking aid were unable to perform a sit-to-stand transfer without using their upper limbs. It is clinically recognized that the addition of technical assistance (such as grab bars, floor to ceilings posts, elevated chairs, etc.) at home for people with ARSACS is necessary to ensure the execution of transfers from sitting to standing or to make these transfers safer.

The decrease in walking speed, combined with the difficulty to perform a sit-to-stand transfer, is also reflected in the performance of the TUG test. Indeed, the time required to perform this task is greater than those of people who are over 89 years-old [48]. People who use a walking aid perform this task much slower than those without a walking aid. These results are congruent with those obtained in the tests measuring the walking speed as well as the transfer from sitting to standing. As reported in the literature, people with hereditary ataxia take longer to change direction despite using compensatory strategies [49]. Since changes in direction are performed on a daily basis, it would be relevant to quantify and describe the execution of this task in ARSACS population in a future study so that specific interventions can be developed [50].

The poor performances achieved in all tests by the cohort of walkers with ARSACS in this study as compared to reference values confirm the early and significant impairments affecting lower limb functions, balance and mobility in this population. Balance and mobility tests’ results are more highly correlated with disease severity than with age, which reflects the age variability regarding the progression of the disease. The results also show a strong association between the severity of the disease and the level of physical performance in mobility activities (r > 0.760), which suggests that the progression of signs and symptoms associated with the disease may be linked to mobility issues. This association was also demonstrated in different hereditary ataxias (r = 0.576) [13].

There are some limitations to this study. Although this study was performed on the largest cohort to date of adult walkers with ARSACS, the number of participants was small. There is also the homogeneity of our sample, which may limit the generalizability of our findings to other ARSACS populations where the specific mutation causes only partial protein production as opposed to a complete absence of protein production in our population [51]. Lower limbs peripheral nerve conduction assessment of the lower limbs would have been interesting in order to quantify the neuropathic involvement and its potential relationship to indoor mobility. In addition, indoor mobility activities were evaluated in a controlled environment, which restrains the generalization of our results to activities that would have been performed at home in a more ecological environment.

## Conclusions

This is the first study to focus on adult walkers with ARSACS. Our results showed that this population has a high level of lower limb impairments and activity limitations despite their young age. One of the most important finding of this study is the high level of lower limb muscle cocontraction during knee movement. The extent of early aging in terms of the ability to carry out daily activities was highlighted. Longitudinal studies are warranted to identify outcome measures that could be considered as biomarkers of the disease progression in future clinical trials. Finally, results of this study could help target the impairments and activities for which prevention and rehabilitation should focus on.

## Data Availability

The datasets used and/or analysed during the current study are available from the corresponding author on reasonable request.
